# Subcapsular Hepatic Hematoma After Endoscopic Retrograde Cholangiopancreatography: A Case Report and Review of Literature

**DOI:** 10.1097/MD.0000000000001041

**Published:** 2015-07-02

**Authors:** Maurizio Zizzo, Andrea Lanaia, Italo Barbieri, Claudia Zaghi, Stefano Bonilauri

**Affiliations:** From the Department of General Surgery, C.S. General and Emergency Surgery, Azienda Ospedaliera – IRCCS Arcispedale Santa Maria Nuova, Reggio Emilia, Italy.

## Abstract

Endoscopic retrograde cholangiopancreatography (ERCP) is one of the most frequently performed procedures for the diagnosis and treatment of biliary-pancreatic diseases. ERCP-related complications total around 2.5% to 8%, with a mortality rate ranging from 0.5% to 1%. An exceptional ERCP complication is subcapsular hepatic hematoma, and few cases are reported worldwide.

We present the case of a 52-year-old woman with a history of recurring upper abdominal pain and a clinical and ultrasonographic diagnosis of obstructive jaundice due to common bile duct stones. After 2 difficult endoscopic biliary procedures, common bile duct stones clearance was obtained. Post-ERCP course was symptomatic with upper abdominal pain and anemization with hemodynamic instability.

CT scan demonstrated a 15 cm × 11 cm subcapsular hepatic hematoma filled with air and liquid on the surface of the right hepatic lobe. The patient was successfully treated with the embolization of a small branch of right hepatic artery angiographically identified as the cause of bleeding.

Subcapsular hepatic hematoma after ERCP is a rare complication that must be taken into account in the differential diagnosis of symptomatic cases after ERCP. Its diagnosis is based on clinical and laboratory data and especially on imaging (ultrasound, CT, or MRI). Treatment is often conservative but, in some cases, embolization or percutaneous drainage or surgery may be necessary.

## INTRODUCTION

Nowadays, endoscopic retrograde cholangiopancreatography (ERCP) is one of the minimally invasive most frequently performed procedures in the diagnosis and treatment of biliary-pancreatic diseases. If performed by experienced professionals, complications related to this total around 2.5% to 8%, with a mortality rate ranging from 0.5% to 1%.^1,2^ An exceptional ERCP complication is represented by subcapsular hepatic hematoma. Since 2000, when subcapsular hepatic hematoma was first described,^3^ world literature has not reported more than 20 cases.^2–19^ In this case report, we describe: a further rare case of subcapsular hepatic hematoma manifested only with abdominal pain as first symptom, in accordance with most clinical presentations of similar cases already described; an analysis of the possible pathophysiological mechanism and a review of present research literature.

## CASE PRESENTATION

We introduce the case of a 52-year-old woman with a history of recurring upper abdominal pain; the last episode required hospitalization after a diagnosis of obstructive jaundice by common bile duct stones. The patient presented jaundice, increase of cholestasis indices, hyperbilirubinemia (mainly direct), ultrasonographic finding of numerous gallstones, and ectasia of the common bile duct to the distal portion, where several gallstones were observed. She had a negative personal history for noteworthy medical and surgical diseases, previous surgery, hemostasis disorders or spontaneous bleeding. Her laboratory tests showed white blood cells 4.5 × 1.000/μL (4–10 × 1.000/μL), red blood cells 4.03 × million/μL (4.3–5.5 × million/μL), hemoglobin 12.9 g/dL (12.5–15.5 g/dL), platelets 192 × 1.000/μL (150–450 × 1.000/μL), bilirubin 6.9 mg/dL (0.3–1.2 mg/dL), aspartate aminotransferase 144 U/L (2–40 U/L), alanine aminotransferase 164 U/L (4–49 U/L), prothrombin time ratio 1.02 (0.8–1.2), international normalized ratio 1.02 (0.8–1.2), and activated partial thromboplastin time ratio 1.16 (0.8–1.2). As a preliminary procedure to subsequent laparoscopic cholecystectomy, the patient underwent a biliary drainage by endoscopic biliary sphincterotomy performed on a 0.035 inch diameter, 450 cm length straight-tip guidewire (producer brand). This rather complicated procedure led to the extraction of multiple small common bile duct stones, but the clearance could not be completed because of respiratory problems in patient that allowed only the placement of a nasobiliary tube. On the first day, a trans-nasobiliary tube cholangiography was performed; as a result, in addition to the tube's endpoint, located in an intrahepatic branch of the right lobe, another 2 massive common bile duct stones were detected. On the second day, a second endoscopic operational procedure was performed and it turned out difficult too; through this procedure, a complete clearance of common bile duct was achieved. At 24 hours from this procedure, patient began complaining of intense mainly upper abdominal pain, radiating to the right shoulder region, with no peritonism or other signs or symptoms, and pain medication was not working; laboratory tests revealed 2.1 g/dL bilirubin (1.6 direct g/dL), aspartate aminotransferase 73 U/L (2–40 U/L), alanine aminotransferase 87 U/L (4–49 U/L), C-reactive protein 9.15 mg/dL (0–0.5 mg/dL), other parameters were within normal limits (including serum amylase). At 36 hours the clinical picture progressed to hemodynamic shock with hypotension, anuria, and hemoglobin levels declining from 11.7 to 8.4 g/dL (12.5–15.5 mg/L). She was treated with transfusion of packed red blood cells and plasma, as well as intravenous administration of crystalloids and antibiotics.

**FIGURE 1:**
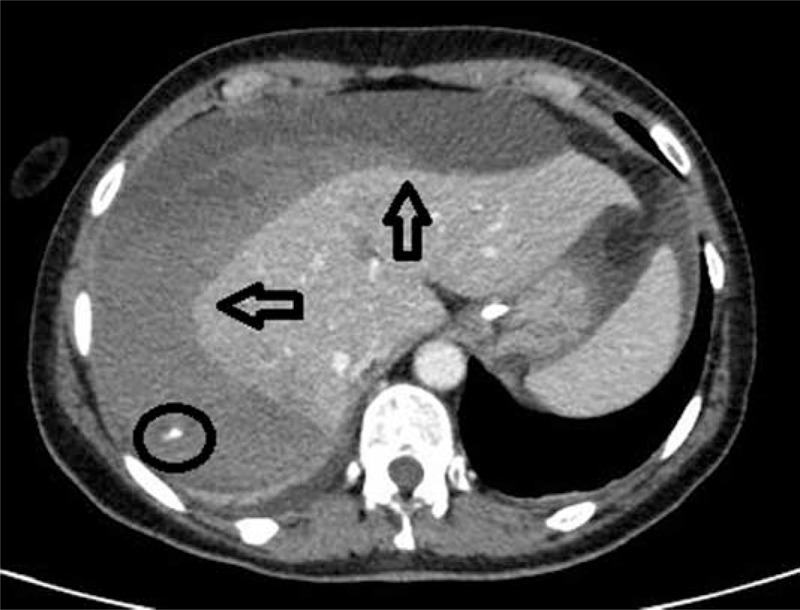
CT scan of upper abdomen showing a subcapsular hepatic hematoma (hypodense area indicated by the arrows) in which is present a source of active bleeding (circle). CT = computed tomography.

**FIGURE 2:**
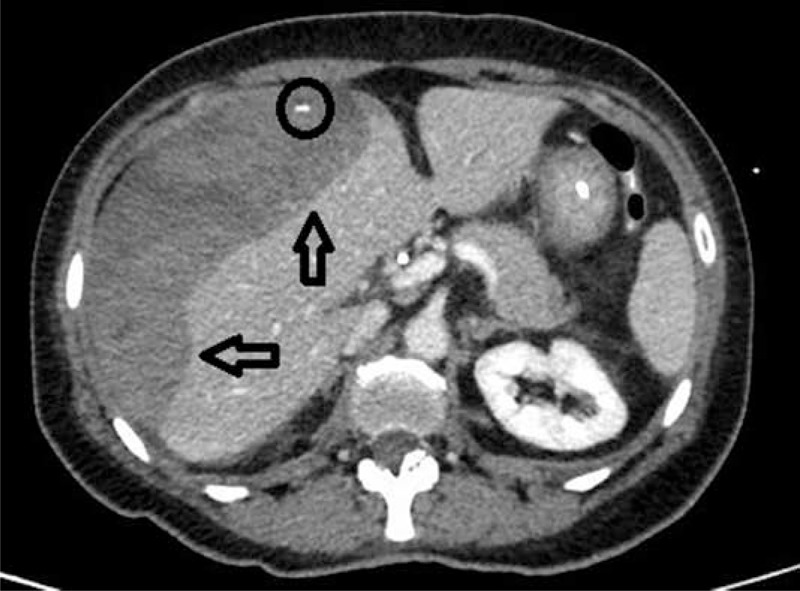
CT scan of middle abdomen showing a subcapsular hepatic hematoma on the surface of the right lobe of liver (hypodense area indicated by the arrows) in which is present a source of active bleeding (circle). CT = computed tomography.

**FIGURE 3:**
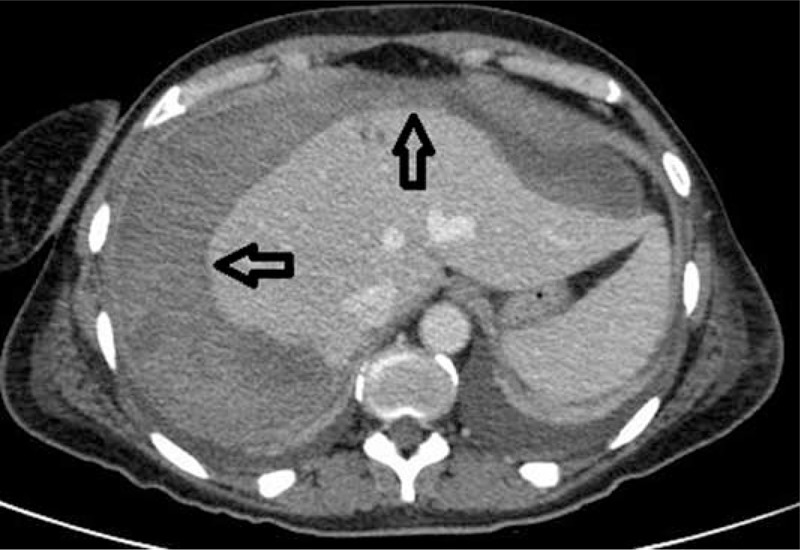
CT scan of upper abdomen showing a reduction of subcapsular hepatic hematoma (hypodense area indicated by the arrows), mainly on the surface of the right lobe of liver. There are no sources of active bleeding. CT = computed tomography.

Urgent computed tomography (CT) scan demonstrated a 15 cm × 11 cm subcapsular hepatic hematoma filled with air and liquid on the surface of the right hepatic lobe (Figures 1 and 2). Urgent angiography was performed with evidence of active bleeding starting from a small branch of right hepatic artery, which was selectively embolized. The postembolization course was regular and hemodynamically stable, without fever, without abdominal pain, and the resumption of normal hemoglobin values during the following week. During a follow-up abdominal CT, performed 10 days after embolization, a marked reduction of hematoma was observed (Figure 3).

## DISCUSSION

A clinically significant bleeding complication associated with ERCP and sphincterotomy records an approximately 2% rate, according to the different cases, although an endoscopically visible but not clinically detectable bleeding is more commonly detected, ranging from 10% to 30% of all sphincterotomies.^19,20^ By skilled experienced hands, bleeding or hematoma associated with ERCP and involving liver, spleen, intestinal wall, or abdominal cavity turn out to be an extremely rare but potentially serious event which requires prompt recognition and treatment. Subcapsular hepatic hematoma represents an exceptional event, of which world literature described 21 cases (Table 1). Etiology was not completely clarified, although, in accordance with most authors,^2,19^ subcapsular hepatic hematoma would rather seem the result of small-caliber intrahepatic vessels breaking which is determined by guide wire during endoscopy procedure; hereafter, blood filtration through nip parenchymal liver in centrifugal direction and the presence of a solid capsule would complete pathophysiology. This would justify the presence of air in the hematoma and frequent infections due to the use of a guide wire with a nonsterile technique. After a detailed analysis of worldwide studies and, particularly, in our case, we too came to the conclusion that subcapsular hepatic hematoma stems from accidental puncture by guidewire of intrahepatic biliary tree and resulting breakage of small caliber intrahepatic vessels.

**TABLE 1:**
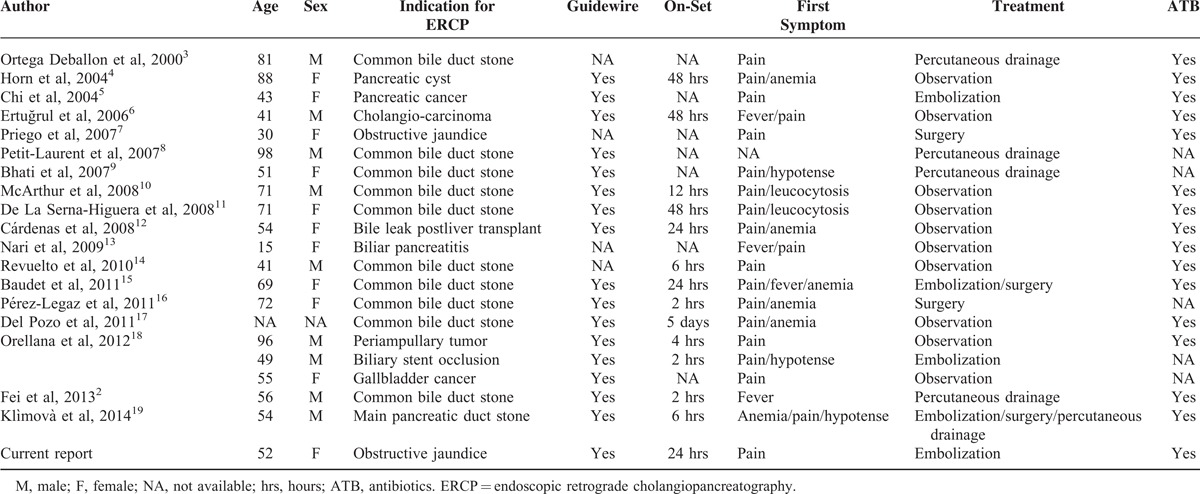
Patient Characteristics From the Reports of Subcapsular Hepatic Hematoma Post-ERCP

Analyzing data in previous report cases, 86% cases report abdominal pain as first clinical expression, often associated with anemia (28.6%) and fever (19%). The onset of these only symptoms, in association with immediate or subsequent hypotension, should suggest the presence of subcapsular hepatic hematoma. However, in agreement with most authors, the incidence of this complication might be underestimated, as most patients might show no symptoms and instrumental post-ERCP monitoring is uncommon.^4,19^ Under no circumstances can laboratory tests be considered major indicators of subcapsular hepatic hematoma, with the exception of a decrease in hematocrit and hemoglobin levels; on the other hand, instrumental investigations such as ultrasound, CT, or magnetic resonance imaging are to be considered diagnostic methods.^19^ Most cases are treated conservatively, this represents treatment choice in the case of a patient who is persistently hemodynamically stable. In such cases, we recommend the use of prophylactic antibiotics, given the risk of infected hematoma.^17^ Surgical treatment should be taken into account in case of deterioration of general clinical conditions, hemodynamic instability, infected hematoma, or high risk of hematoma breakage.^17^ This treatment consists in draining hematoma, hemostasis if possible, and follow-up by CT, in order to monitor progress.^19^ A viable alternative to surgical treatment is represented by selective or superselective embolization of involved vessels or percutaneous drainage of hematoma. In the cases reported, (Table 1) with reference to the needs of different kinds of treatment in some cases, 47.6% patients were treated conservatively, 23.8% patients with percutaneous drainage, 23.8% patients with embolization, and 19% patients required surgery. In our case, hemodynamic stability regained only with infusion therapy and angiographic findings of active bleeding led us to choose treatment by embolization, with resolution of clinical picture. To conclude subcapsular hepatic hematoma after ERCP is a rare and alarming complication that must be known and taken into account in the differential diagnosis of symptomatic cases after ERCP. Its diagnosis is based on clinical data (abdominal pain, anemia, hypotension, and fever), laboratory data (decrease in hematocrit and hemoglobin levels), and especially on imaging (ultrasound, CT, or magnetic resonance imaging).^7,13^ Treatment is often conservative but, in some cases, embolization or percutaneous drainage or surgery turn out to be necessary.
